# SpaceTime-SonoNet: efficient classification of ultra-sound video sequences

**DOI:** 10.1007/s11517-025-03504-w

**Published:** 2026-01-31

**Authors:** Matteo Interlando, Luca Zini, Nicola Guraschi, Nicoletta Noceti, Francesca Odone

**Affiliations:** 1 MaLGa-DIBRIS, Università degli Studi di Genova, Genova, Italy; 2 Esaote SpA, Genova, Italy

**Keywords:** Scan-plane detection, Ultra-sound, Efficiency, Deep learning

## Abstract

**Abstract:**

In this paper, we extend the SonoNet architecture to capture spatio-temporal information from ultra-sound (US) sequences. More specifically, we propose 3D-SonoNet32 – which lifts 2D convolutions to 3D – and to an efficient (2+1)D variant – to keep the computational cost under control while preserving the benefits of the spatio-temporal model. We investigate the potential of these architectures on a scan-plane detection problem and discuss how these methodologies can be beneficial for AI-driven online “scan assistants”, to enhance the quality and reproducibility of the evaluation and ultimately support the clinicians in the US examination. Our main contributions are (i) the design of novel Space-Time SonoNet architectures for analysing US video sequences, (ii) an in depth experimental analysis to show the benefit of using space-time models with respect to purely spatial ones, and to discuss the potential improvements gained by exploiting domain-specific properties like temporal coherence and prior knowledge of the ongoing scan. Overall, we show that the proposed models are specifically designed to be computationally lightweight, but also competitive in performance, making them suitable for real-time deployment on portable US devices.

**Graphical abstract:**

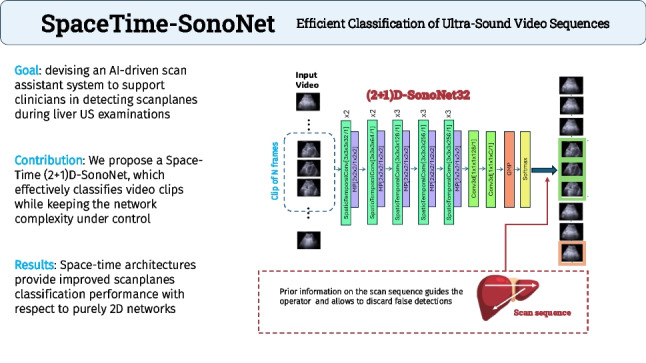

## Introduction

Ultra-sound (US) imaging plays a fundamental role in clinical practice due to its non-invasiveness, cost-effectiveness, and real-time capabilities. An important aspect of US examinations is the identification of Standard Planes (SPs), which are US images characterized by the presence of specific landmarks, relevant for the clinical examination. Unfortunately, the identification and interpretation of SPs requires specific expertize and remain highly operator-dependent, often leading to significant inter-observer and intra-observer variability [1]. More specifically, in the context of liver scanning – a critical diagnostic procedure for assessing hepatic health – these limitations can affect diagnostic accuracy and completeness of organ coverage. In fact, several technical challenges contribute to the difficulty of liver US scanning [2–4]- The anatomical position of the liver, partially obscured by the ribs and lungs, can hinder optimal visualisation. Patient-related factors such as obesity and the presence of subcutaneous adipose tissue can attenuate the US signal, reducing image quality. Additionally, intestinal gas can cause acoustic interference, while small or deep-seated lesions may remain undetected. Most importantly, the overall image quality and diagnostic utility heavily rely on the operator’s expertise in selecting scanning parameters, probe orientation, and anatomical plane acquisition.

From all these observations, the necessity of a more objective procedure for SPs detection clearly emerges. Recent advances in Artificial Intelligence (AI) offer promising solutions [5–8], demonstrating the ability to assist in real-time recognition of anatomical structures in US imaging, even on portable devices with limited computational resources. AI algorithms can enhance the objectivity, reproducibility, and completeness of US examinations by guiding the operator during image acquisition.

**Fig. 1:**
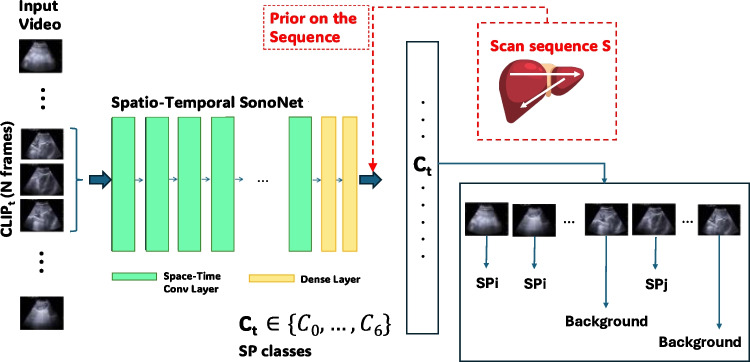
A visual sketch of our pipeline. A video is segmented into clips of N frames, provided in input to a spatio-temporal architecture to detect the presence of scan planes over time. When possible, prior knowledge on the scan sequence is exploited

This study contribute to the implementation of an AI-driven “scan assistant" system to support clinicians during liver US examinations (see Fig. 1). In this context, the primary aim of this paper is to propose a scan-plane detection methodology exploiting the availability of image sequences. The rationale is that during an US examination image sequences are available and may bring additional information and an increased robustness. Specifically, we consider US analysis of the liver which is particularly challenging and is usually carried out with multiple scans following a set of pre-defined paths [9]: these procedure helps the physician to obtain a good coverage of the organ. The paths may be described as sequences of anatomical landmarks; for this reason, scan-plane detection can be also used to assess the full coverage, and thus the completeness, of the examination.

Our study also explores the feasibility of an AI-driven online “scan assistant", to support clinicians during liver US examinations (see Fig. 1). For its applicability in the real-world, it is essential the model is not only accurate, but also efficient in terms of computational cost and parameter count. This is particularly relevant when considering deployment in real-time systems or on portable US devices with limited processing capabilities (as a general reference, those of a normal laptop).

For all these reasons, similarly to our previous research [10], we resort to the SonoNet family [11]. In order to capture the essence of image sequences, we propose an extension to space-time data, proposing a novel 3D-SonoNet, where 2D convolutions are substituted by 3D convolutions. To keep the size of the network under control, we also propose an efficient (2+1)D variant, where a 3D convolution is substituted by a combination of 2D followed by a 1D convolution.

The contributions of the paper can be summarised as follows:

- Novel space-time SonoNet architectures, including a 3D and a (2+1)D version, the latter allowing us to keep the number of parameters under control
- The application of such architectures to standard scan-plane detection for the efficient analysis of liver US sequences
- An experimental analysis discussing the potential improvements one could obtain if exploiting specific properties of the application domain, including temporal coherence and a prior on the specific scan under process.

The remainder of this paper is organised as follows. Section 2 reviews related work. Section 3 details the proposed methodology, including 3D SonoNet and its (2+1)D variant. Section 4 presents the experimental assessment, evaluating the performance of the system through quantitative metrics. Finally, Section 5 is left to conclusions.

## Related works

Existing methods for automatic detection of standard scan-planes in US data leverage either spatial information alone [11–16] or both spatial and temporal information [17–19]. The SonoNet family, at the basis of our work, was first introduced in [11] to address real-time automatic detection of fetal standard views in 2D US data. SonoNet is obtained from VGG16 [20] by replacing fully connected layers with $1 \times 1$ convolutional filters. In [12], the authors improved SonoNet by incorporating a self-gated soft-attention mechanism. They aimed to overcome a limitation in SonoNet’s ability to capture local information, which is crucial for differentiating similar anatomical views, such as cardiac planes. Attention maps are also generated in [14], where the authors propose SonoEyeNet, a multi-task Convolutional Neural Network (CNN). They adopt a generative mechanism to mimic a sonographer’s visual attention to guide and help the classification. A transfer learning approach is adopted in [13], where fetal scanplane detection is addressed in the specific scenario of limited data availability. The authors transfer a CNN pre-trained on ImageNet, later fine-tuned on fetal US frames extracted from videos. Despite the presence of videos, time is not explicitly exploited in this work. In [15], the authors investigated the use of contrastive learning, specifically employing SimCLR as a pre-training strategy, to reduce the dependency on large annotated US datasets for fetal standard plane classification. They evaluated both scenarios where the backbone was initialised with ImageNet weights and where it was trained in an end-to-end dual-task setup. Their study focused on analysing how classification performance is affected by varying the amount of labelled training data. In [16], the authors proposed an ensemble approach combining multiple CNN architectures—AlexNet, VGG-19, and DarkNet-19—to classify fetal standard planes. The final prediction was obtained using an absolute voting scheme over outputs from a softmax and a random forest classifier. While this ensemble improves classification performance, it significantly increases computational complexity, posing challenges for real-time deployment on lightweight or resource-constrained US devices.

More related to our work are approaches that adopt temporal strategies to address SP detection. [17] proposed a knowledge-transferred recurrent neural network exploiting spatio-temporal information. It is essentially a hybrid multi-task model combining CNNs and Long Short-Term Memory (LSTM) networks that act on short video clips. Their experiments demonstrated the advantages of multi-task knowledge transfer, showing the gain provided by explicit temporal information compared to models that only used spatial features. A comparison between different strategies to exploit the time component is discussed in [19], where RGB video clips are combined with optical flow in a multi-stream architecture. The work [18] advances [14] by introducing a temporal visual attention that produces dynamic attention maps for each frame. The core architecture is a bidirectional Recurrent Netural Network (RNN), surpassing the results of the 2D SonoNet model [11].

In the context of SP classification, only a limited number of studies have exploited both spatial and temporal information from US videos, but the gain of using time information has been assessed more generally. For instance, breast US video classification has been addressed in [21], where 3D ResNet is shown to be superior to the 2D model. [22] proposes a dual-branch classification strategy that combines a region-guided and a time-guided module. The latter simulates radiologists’ temporal attention in the diagnosis process as a guiding message, helping the network focus on frames that are more beneficial to the diagnosis. In [23], the authors address lung US video classification to diagnose COVID-19. An LSTM model captures temporal dependence.

From the works above, we may conclude that despite the differences in adopted approaches and addressed tasks, the benefit of exploiting time information – when available – for solving US classification or detection has been consistently observed. This guided us in this direction to solve our new SP detection task. Today, the gold standard for modelling sequences is the Transformer [24] that, despite its effectiveness, is still too demanding from a computational standpoint to be considered in scenarios where efficiency is essential, as in our case. Considering the fact that SonoNet is still a reference 2D network for US tasks that provides a good compromise between efficiency and accuracy, a more natural approach has been to reason on its extension to 3D convolutions – to the best of our knowledge, the first attempt in this sense. However, lifting the original architecture from 2D to 3D brings an increased computational burden that we addressed by decoupling 3D in (2+1)D convolutions, following [25].

**Fig. 2:**
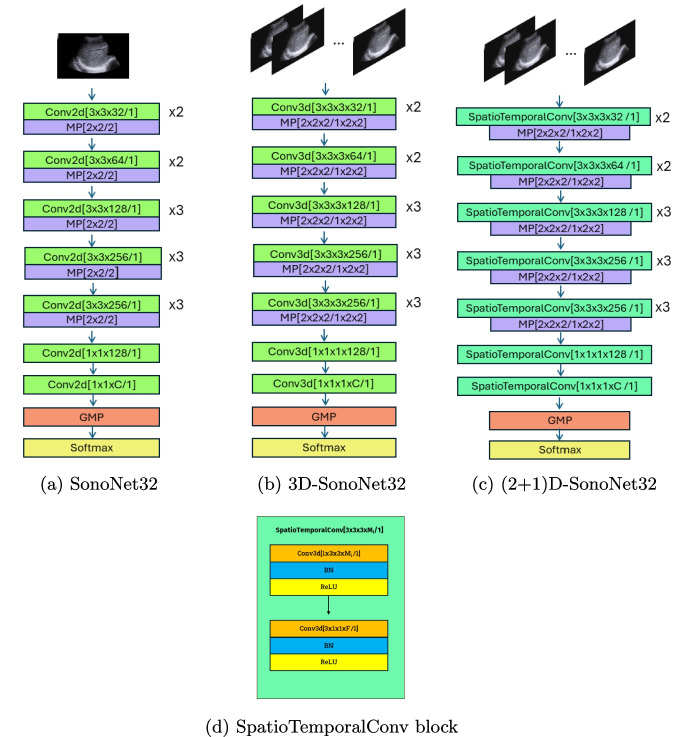
The three architectures adopted in this work. Figure 2a represents the original SonoNet32 applied to single 2D US frames. In Fig. 2b we report our proposed 3D-SonoNet32, designed to process clips of US frames. Finally, Fig. 2c shows a (2+1)D variant of the 3D SonoNet32. Details about the SpatioTemporalConv layer are shown in Fig. 2d. In the squared brackets, we report details of the convolutional operation: [$\text {kernel size} \times \text {number of kernels}$/stride]. The kernel size includes temporal extent, height, and width

## Proposed architecture

The baseline architecture we start from is SonoNet [11], which was proposed specifically for US classification and in its original formulation has been inspired by the VGG16 model [26] (see Fig. 2a). It consists of two main components: a feature extractor module and an adaption module. The feature extractor module includes 17 layers, with 13 convolutional layers and 4 max-pooling layers. Each convolutional layer is followed by batch normalisation; after each max-pooling, the number of filters is doubled. In the adaption module, the fully connected layers of the VGG16 architecture were replaced with two $1 \times 1$ convolutional layers. The number of filters is then reduced to match the number of target classes C. Hence, the output of the adaption module results in a C-class score map with higher spatial resolution. These are subsequently flattened through an average-pooling layer and fed into the softmax activation.

Different variants of the SonoNet have been proposed by changing the number of filters in the first convolution layer. In our study, we opt for the SonoNet32 variant, which has been shown to balance accuracy and computation [11].

While SonoNet32 efficiently captures spatial information from US images, the dynamic nature of US exams, where anatomical structures shift and change across frames, opens the possibility of incorporating temporal information as well. For example, detecting the presence of the heart in a single US frame can be challenging due to variability in the visibility of the organ in a static image. However, when consecutive frames are analysed, the rhythmic motion of the heartbeat becomes evident, greatly improving heart detection accuracy. Thus, the integration of spatial and temporal information is essential for more accurate standard plane classification.

To address this, we propose to extend the 2D SonoNet32 architecture to 3D, to leverage the temporal dimension by processing clips of consecutive frames rather than isolated 2D images. This allows the model to learn spatial features within each frame and temporal patterns across frames, enhancing the robustness of the classification.

Our first contribution is 3D-SonoNet32 (see Fig. 2b), which builds upon the 2D version by replacing 2D convolutional and 2D max-pooling layers with 3D convolutions and 3D max-pooling layers. This enables the model to capture spatio-temporal features from the input volumes of frames.

On one side, this straightforward extension from 2D to 3D extends the network expressivity, but on the other, it also causes a significant increase in the number of parameters (of approximately one order of magnitude). For this reason, inspired by the works [25] and [27] which considered the ResNet family, our second contribution is (2+1)D-SonoNet32 (see Fig. 2c), in which we factorise the 3D convolutions of our 3D-SonoNet32 into separate 2D spatial and 1D temporal convolutions. More specifically, each 3D convolution is replaced by a SpatioTemporal block (see Fig. 2d), which applies a 2D convolution across the spatial dimensions of each frame, followed by a 1D convolution along the temporal dimension. To this purpose, a 3D convolutional kernel of size $k_t \times k_h \times k_w$ – where $k_t$, $k_h$, and $k_w$ corresponds to the temporal extent, height, and width of the filter respectively – is factorized into a 2D spatial convolution of size $1 \times k_h \times k_w$, followed by a 1D temporal convolution of size $k_t \times 1 \times 1$. We also decompose the strides to handle spatial and temporal dimensions separately, ensuring efficient processing of frame sequences: we set a stride of (0, 1, 1) and (1, 0, 0) for the spatial and temporal convolutions, respectively.

Between the spatial and temporal convolution was placed a ReLU activation, effectively doubling the model’s non-linearity operations without increasing the number of parameters.

Table 1 provides a comparison of the number of parameters and the computational cost (in Giga-operations) for each model configuration. Notice how in our (2+1)D variant we keep the size of the network under control, making it comparable to the 2D counterpart on the number of parameters and, in any case, halving the figures with respect to the 3D variant. We also report the size of a (2+1)D version where the numbers of convolutional kernels are adjusted to approximate the number of parameters of a fully 3D network. This variant, marked with an *, has been derived following [25].

**Table 1:** Comparison of the number of parameters and computational cost (in Giga-FLOPS) for the different architectures of the SonoNet family used in this study. We mark with (*) a configuration of the (2+1)D architecture where the number of parameters is approximately the one of the 3D version, which we derived following [25]

Model	# Parameters	G-FLOPS
SonoNet16 (2D)	930334	20.12
SonoNet32 (2D)	3715630	80.17
SonoNet64 (2D)	14851150	320.05
3D-SonoNet32	11070574	954.40
(2+1)D-SonoNet32	5094381	459.54
(2+1)D-SonoNet32*	11081431	774.34

## Experimental analysis

In this section, we discuss the experiments we conducted. After an introduction of the data, we will provide details about our experimental protocol and finally discuss the quantitative analysis we performed for the classification tasks.

### Raw data

Considering the unavailability of US video benchmarks in public datasets, our analysis is based on a private, anonymised US dataset of the abdominal body area, henceforth called US-DATASET. It includes 2086 videos of 413 patients, for a total of 388787 frames. For most patients, we have the complete exam, which consists of 5 different video scans following different paths. Within the dataset, 6 types of standard scan-planes have been annotated: 0.*Portal Bifurcation*, 1.*Hepatic Vein*, 2.*Gallbladder*, 3.*Heart*, 4.*Kidney*, 5.*Ilo*, plus an additional class with background views that do not refer to any standard plane (the class 6.*Other*). Data labeling was carried out in two steps: the first one, carried out by radiologists was performed with the Intel CVAT tool^1^, and the annotators applied bounding boxes around the individual anatomical structures. The second step, carried out by Machine Learning experts supervised by radiologists, consisted in associating a semantic label describing a scan-plane of interest, following a set of if-then rules.

The classes are characterised by an uneven complexity, since some of them are defined by the simultaneous presence of different anatomical structures while for others the presence of a single structure is enough. For instance, while class 1 requires the presence of both the right and left branches of the portal vein, class 5 is only defined by the presence of ilo. Hence, not only the number of structures in the two planes is different, but also their spatial extent can vary, leading to classes characterized by an uneven amount of informative content.

### Data preparation and split

We extract training, validation and test splits according to the following considerations. The $15\%$ of complete (i.e. including all 5 video scans) exams are used to form the test set. The completeness of the test allows us to explore different strategies at inference time. The remaining $85\%$ of videos

were assigned to the training set. This resulted in a test set of 280 videos from 56 patients, and a training set containing 1806 videos of 357 patients. For each training-validation split, we follow a similar procedure, with a proportion of $80-20\%$ in this case. Once the procedure is complete, each patient can not appear in more than one sets, ensuring a fair evaluation.

Both the frame-by-frame (2D) and spatio-temporal (3D) experiments were conducted using the same training, validation, and test video splits. For a fair comparison, we produce sets of the same size in the 2D and 3D variants. To this purpose, we first produced the dataset for the 3D model by extracting clips of non-overlapping N consecutive frames (N being a hyperparameter of the method), each of which was associated with the same ground truth label. This ensures that the anatomical structures and SP remain consistent throughout the clip.

Based on the 3D (video clips) dataset, we built the 2D one by simply selecting the central frame of each clip. This ensures that the frame is always significant (since the clip has been built with coherent labels) and the different models compete on datasets of comparable complexities, emphasising the role of the time component.

The dataset presents significant class imbalance. Indeed, the cardinality of the training set is as follow: 0.*Portal Bifurcation* $\rightarrow$ 1168, 1.*Hepatic Vein* $\rightarrow$ 3362, 2.*Gallbladder*$\rightarrow$ 768, 3.*Heart*$\rightarrow$ 3244, 4.*Kidney*$\rightarrow$ 4381, 5.*Ilo*$\rightarrow$ 643, 6.*Other*$\rightarrow$ 12428 (the class distribution in the test set follows a similar proportion). The majority of samples in the training set in class 6.*Other*(about $47.8\%$), and a few classes underrepresented (0.*Portal Bifurcation*, 2.*Gallbladder*, and 5.*Ilo*, with respectively, $4.5\%$, $2.9\%$ and $2.5\%$ of samples of the training set). Despite representing a challenge for the learning problem, this imbalance is instrinsic in the nature of the addressed problem, since during the acquisitions some scanplanes are more frequently observed than others.

### Implementation details

In this section, we report implementation details to help reproducibility^2^. The implementation of 2D SonoNet32 is from a publicly available GitHub repository^3^. We adapt the existing resource for our specific use case using the PyTorch framework (specifically, our implementation adapts SonoNet32 to process grayscale US images). Python was used to implement the methodology and integrate the model into our workflow.

To attenuate the issue of class imbalance, we adopt two common strategies. First, after normalisation to scale the pixel values between 0 and 1, we employed a data augmentation technique for the training images. This includes vertical and horizontal flips with a probability of $50\%$, gamma correction with randomly selected gamma value between 0.7 and 1.4, a custom resize with scale factor randomly selected between 0.8 and 1.2, and random rotation between $-10^\circ$ and $+10^\circ$. These augmentations help to artificially increase the diversity of the training data and mitigate the overfitting issue by providing more varied training examples, as supported by previous studies [28, 29].

In addition to data augmentation, we utilised a random weighted sampler to ensure that each training batch contained approximately the same number of samples from each class. This approach helps to address the class imbalance (see Section 4.2) by giving underrepresented classes more visibility during training. By doing so, we ensure that the model does not become biased towards the majority class, promoting better generalisation and balanced performance across classes [30].

The batch size used during the training was 128 for the 2D approach and 8 for the 3D approaches (for computational constraints). Each model is trained for a maximum of 200 epochs, with an Adam optimiser and an early stopping criterion on the validation loss. The early stopping patience was set to 10 epochs, allowing the training to stop if no further improvement on the validation loss. The learning rate is adjusted using a *R*educeLROnPlateau scheduler. The initial learning rate value was set to $1e^{-5}$ for the 2D and $1e^{-4}$ for the 3D approach. To further mitigate overfitting, we incorporated weight decay regularisation of $1e^{-4}$ into the Adam optimiser. Weight decay penalises large weights, promoting simpler models, reducing overfitting, and hence improving generalisation to unseen data [31].

To assess the robustness and reproducibility of the implemented methods, we adopt a hold-out cross-validation procedure consisting of three independent data splits. Each split is generated using a different random seed to ensure reproducibility, while maintaining the same 80–20 % training–validation ratio described above. The test set is kept fixed across all runs, so that only the training and validation partitions vary. The results reported in Sections 4.4 are averaged over the three folds.

All experiments were conducted on a single NVIDIA A30 GPU with 24 GB of RAM.

### Results

This section reports the results of our method on the US-DATASET, including a comparison between the different architectures.

#### Baseline 2D scan-plane detection

We begin by presenting some baseline results. To determine the optimal network size, we conducted a preliminary experiment on a single data split built from the whole set of data we have available. We compared different variants of the SonoNet architecture, trained from scratch: SonoNet16, SonoNet32, and SonoNet64. Our results showed that performance generally improved with larger architectures. Specifically, SonoNet64 achieved the highest test accuracy at $53.82\%$, compared to $52.79\%$ for SonoNet32, and $51.05\%$ for SonoNet16. Although SonoNet64 outperformed the others, the improvement over SonoNet32 was marginal, while the number of trainable parameters increased significantly (by approximately 299%), see Table 1. Therefore, for the subsequent experiments, we selected SonoNet32 as a trade-off between performance and model complexity.

To mitigate overfitting, we applied weight decay regularisation with a coefficient of $1e^{-4}$. This resulted in an improvement over the previous SonoNet32 performance, increasing the test accuracy to $54.65\%$. Given this benefit, weight decay regularisation was employed in all subsequent experiments.

Considering this last network configuration, Table 2 reports the average results we obtain over three training-validation hold-outs. Besides training the architecture from scratch, we also consider the possibility of using a pretrained model: we adopt a transfer learning strategy, pretraining the model^4^ on the fetal US dataset introduced by [11] before fine-tuning the model on our dataset. As expected, the pre-training helps to improve the results with a $+3.58\%$ of accuracy on the test. This may be explained with the better generalization ability of a pre-trained model.

**Table 2:** 2D baselines on the entire frame-by-frame dataset. We report mean test accuracy and standard deviation obtained with a 2D-SonoNet32 over three training-validation hold-outs

Model	Test accuracy (%) ± std
(2D) SonoNet32 trained from scratch	53.31 ± 0.95
(2D) SonoNet32 with pretraining	56.89 ± 1.59

#### The benefits of a spatio-temporal input

When extending our analysis to spatio-temporal data, for a fair comparison between 2D and 3D models, we rely on the datasets of the same cardinality derived as discussed in Section 4.2.

We start by discussing the choice of an appropriate N, i.e. the length of the input clip. To this purpose, we initially carried out a preliminary analysis, comparing the results obtained with N=5 and N=10. This choice was motivated by a trade-off between capturing sufficient temporal information and keeping the computational cost manageable. We observed consistently higher results when N=10, with a gain on average of $+1.15\%$ with respect to the use of shorter clips. This improvement is likely due to the increased temporal information available in longer clips, which allows for better feature extraction and more accurate representation of the dynamic processes in the data. Hence, we adopt N=10 as a reference choice in our experiments.

Table 3 reports the results obtained by the two frame-by-frame variants of the model (trained from scratch and pre-trained on the US fetal dataset) and by a 3D variant (3D-SonoNet32) trained from scratch only. We do not report a pre-trained 3D variant since no US video benchmark is available for an initial pre-training. Despite this disadvantage, the 3D model significantly outperformed the 2D variants, achieving higher accuracy and lower standard deviation. This result highlights the advantage of jointly leveraging both spatial and temporal information in the learning process. This is at the price of a higher computational cost due to an increased number of parameters (see Table 1). In the next section, we will evaluate the robustness of the (2+1)D model while keeping the computational burden under control.

**Table 3:** Comparative analysis between frame-by-frame (2D) and spatio-temporal (3D) versions, trained on the subsampled dataset described in Section 4.2. Test accuracy and standard deviation are averaged over three training-validation hold-outs

Model	Test accuracy (%) ± std
(2D) SonoNet32 trained from scratch	53.23 ± 4.39
(2D) SonoNet32 with pre-training	57.73 ± 1.56
3D-SonoNet32	63.58 ± 1.02

#### Tradeoff between accuracy and complexity of the network

While the benefits of adopting a spatio-temporal input have been shown in Table 3, we recall how Table 1 reported an increase in the number of parameters of nearly 300%. Table 4 reports the results with three variants of the implementation of the (2+1)D architecture, which we designed to control the network complexity while retaining the temporal information. In a first experiment, we consider a (2+1)D version whose size is derived directly from the 3D one, as detailed in Section 3. Our results show that the (2+1)D version is able to produce comparable results to the 3D counterpart while using only about one-third of the parameters, demonstrating a favourable trade-off between accuracy and computational efficiency.

**Table 4:** Comparative study between different versions of (2+1)D-SonoNet32, trained on the sub-sampled dataset described in Section 4.2. Test accuracy and standard deviation are averaged over three training-validation holdouts. (* as in [25], see text for further details)

Model	Test accuracy (%) ± std
(2+1)D-SonoNet32	63.54 ± 0.95
(2+1)D-SonoNet32*	62.14 ± 1.12
(2+1)D-SonoNet32 w/o BN	63.43 ± 1.02

We also implemented a second (2+1)D variant inspired by the approach proposed in [25], which aims to match the parameter count of the original 3D model rather than reduce it. This method was shown to improve performance in the context of ResNet architectures. However, in our case, based on SonoNet, such improvements are not consistently observed, suggesting that the effectiveness of this parameter-balancing strategy may depend on the backbone architecture.

In a further experiment, we carry out an ablation to discuss the usefulness of the intermediate batch normalisation (BN) between the 2D and the 1D convolutional blocks in the (2+1)D decomposition. Our results indicate that while BN brings a slight benefit in terms of stability and accuracy, its overall contribution remains marginal and does not significantly alter the final performance.

**Fig. 3:**
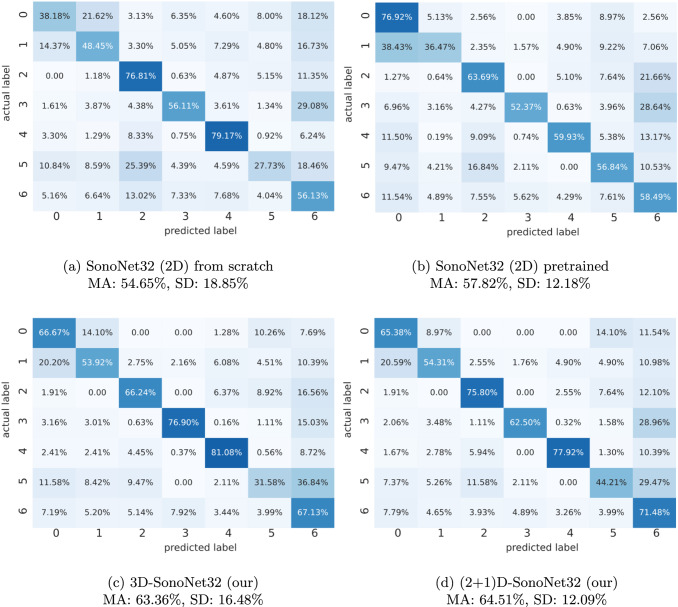
Confusion matrices generated by (2D) SonoNet32 trained from scratch (3a), (2D) SonoNet32 pretrained (3b), our 3D-SonoNet32 (3c), and our (2+1)D-SonoNet32 (3d), showing the classification performance on the US-DATASET test set. MA and SD refer, respectively, to classes’ Mean Accuracies and Standard Deviation

Finally, for a more detailed class-wise comparison, Fig. 3 reports the confusion matrices obtained by a single hold-out on the 2D (both pre-trained and trained from scratch), 3D and (2+1)D models. We may notice an overall improvement in the different classes for spatio-temporal models, with lower confusion among different classes. The inclusion of temporal context leads to fewer misclassifications and greater diagonal dominance in the matrices, indicating improved ability to distinguish among classes. The overall mean accuracy increases from $54.65\%$ (2D from scratch) and $57.82\%$ (2D pre-trained) to $63.36\%$ (3D) and $64.51\%$ ((2+1)D). The lowest standard deviation is of the (2+1)D architecture, comparable to the 2D one with pre-training.

The confusion between similar classes, especially class 0 (*Portal Bifurcation*) and class 1 (*Hepatic Vein*), is a persistent source of error, though it is noticeably reduced in the spatio-temporal models. Similarly, class 5 (*Ilo*) remains the most challenging category, showing consistently lower classification accuracy across the majority of architectures. This difficulty may be attributed to the small size and less distinctive visual features of the *Ilo* region, making it harder to detect and classify reliably – especially when compared to larger and more dynamic structures like the heart or kidney. Nevertheless, the improvement brought by temporal modelling is evident and consistent, confirming its crucial role in enhancing classification performance in US video data.

#### A closer look at the application potential

We conclude our experimental analysis by discussing the specificities of the application domain we are considering. During the acquisition, US image sequences are presented to the operator. In clinical practice, the operator will follow an acquisition protocol where different anatomical landmarks will be visited in a pre-defined ordered sequence. Considering the temporal extent of the data, we first discuss the time coherence of the obtained results, in consideration of the fact that different patients could present more or less challenging conditions.

**Fig. 4:**
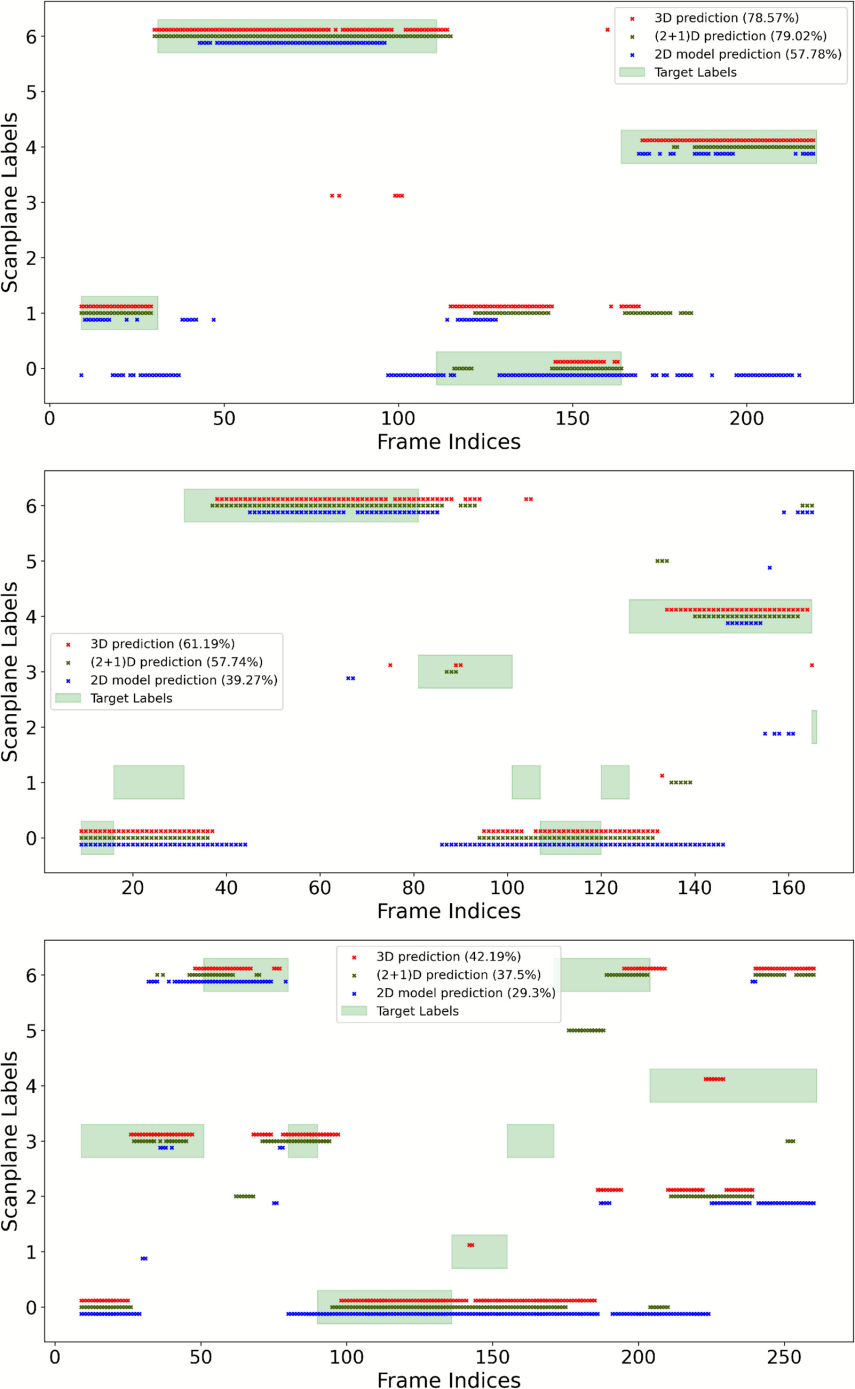
Results obtained on US test videos, comparing the ground truth with the different SonoNet32 architectures we adopted

Figure 4 shows a visual comparison of the predicted labels from the frame-by-frame and spatio-temporal models on US videos, in comparison with the ground truth (green shadow). To mimic a real working scenario, here the test videos have been separated into consecutive clips, which are all considered in the evaluation (regardless of the uniqueness of the label in the clip). This leads to a more challenging test scenario with larger ambiguity in the input samples.

We can notice that both the spatio-temporal models (red and green markers) are generally more coherent over time and closer to the ground truth. In contrast, the frame-by-frame model predictions (blue markers) show fluctuations and more errors. Further, when the labels switch between classes, the frame-by-frame model predictions are inconsistent, switching between different classes, while the spatio-temporal models maintain more stable predictions over consecutive frames.

Table 5 summarises corresponding quantitative results on all the 280 videos included in the test set: here again, the results highlight quite clearly the effectiveness of space-time models. A high standard deviation of accuracies computed on a video basis speaks in favour of the variability of the results from one video to another. The table also reports a comparison with a ResNet derived from [25], with results slightly higher than the ones obtained with our 3D-SonoNet32, at the price of a higher computational cost. Indeed, we report in the table (in parentheses) the number of parameters for each architecture. If we focus on the 3D architectures, we may notice that the ratio between the number of parameters of 3D-SonoNet32 and 3D-ResNet18 is approximately 0.33 with a small reduction in average accuracy of $-2.32\%$. Moving to the (2+1)D models, the trend is even more pronounced: the ratio between (2+1)D-SonoNet32 and (2+1)D-ResNet18 is now about 0.16 with a comparable reduction in average accuracy of $-2.12\%$. Overall, this leads us to conclude that the SonoNet models are more appropriate for the needs and constraints of our target application.

**Table 5:** Average accuracy and standard deviations computed per video on the test set. In the parentheses, we report the order of magnitude of the number of parameters of each architecture (M stands for Millions)

Architecture	2D	3D	(2+1)D
SonoNet32	51.92 ± 18.82	63.09 ± 17.20	62.04 ± 16.80
	($\sim$3.7M)	($\sim$11M)	($\sim$5M)
ResNet18 [25]	62.92 ± 16.55	65.38 ± 16.46	64.16 ± 17.33
	($\sim$11M)	($\sim$33M)	($\sim$31M)

We finally report the improvement that can be obtained if we use the prior on the specific scan of the sequence followed by the protocol (see Fig. 1, red arrow). To carry out this experiment, we train the networks by masking the logits to inhibit classes that are known to be invisible in each scan. As expected, the complexity of the problem reduces, thus all the models obtain a significant improvement. Experiments we carry out on a single training run from scratch allow us to obtain 67% accuracy with a 2D-SonoNet32, 75.45% on a 3D-SonoNet32, and 74.92% with a (2+1)D-SonoNet32.

## Conclusion

In this work, we contributed to the design and implementation of an AI-driven “scan assistant" system to support clinicians during liver US examinations. Specifically, we proposed a methodology for scan-plane detection on image sequences, which we process with extensions of an architecture from the SonoNet family, a reference in this field: we extended SonoNet32 to incorporate the ability to capture spatio-temporal information introducing 3D-SonoNet32 – by moving from 2D to 3D convolutions – and the variant (2+1)D-SonoNet32 – in which we keep the network complexity (number of parameters and G-FLOPS) under control while saving the competitive performance of the 3D counterpart.

The experimental results showed 3D architectures provide better performance compared to 2D solutions. The average accuracy increases from $54.65\%$ of the 2D-SonoNet trained from scratch and $57.82\%$ of the 2D architecture pre-trained on a public dataset, to the $63.36\%$ of the 3D-SonoNet, which further improve with the (2+1)D-SonoNet, reaching $64.51\%$. The latter has also the lowest standard deviation, similar to the one of the 2D one with pre-training. This suggests that spatio-temporal models provide a better generalization ability, similar to the one that can reach on 2D models thanks to the pre-training. Moreover, decoupling 3D convolutions in 2D+1D lowers the computational cost by almost halving the number of parameters.

In a last experimental section, we discussed how to exploit the specificities of the application domain. Specifically, the prior on the acquisition protocol can be used to inhibit the classes that should not appear in that sequence. This allows us to increase the accuracy of spatio-temporal models to $74.92\%$.

Finally we verified the robustness of our method to ambiguity in the input. When using input clips with multiple class labels, spatio-temporal architectures are able to accurately classify them thanks to the temporal context. The average accuracy is $62.04\%$, only $-1.41\%$ with respect to the performance on non-ambiguous clips.

Given its practical nature, our work may be extended in two different directions. The first one refers to improving the interpretability. Given the involvement of clinicians it is important the results can be visually supported to fully understand the meaning of a prediction. In this sense, the use of methods for motion saliency estimation or detection (e.g. [32]) can be an asset. The second direction refers to assessing the efficiency of the scan plane detection pipeline onboard of the acquisition device.

## Footnotes

1 https://www.cvat.ai

2 The code is available at https://github.com/Malga-Vision/SpaceTime-SonoNet

3 https://github.com/rdroste/SonoNet_PyTorch/tree/master

4 Weights are available from https://github.com/rdroste/SonoNet_PyTorch/tree/master
